# Complex post-traumatic stress disorder is linked with accelerated epigenetic aging in female Yazidi ex-captives.

**DOI:** 10.1093/geroni/igaf122.3736

**Published:** 2025-12-31

**Authors:** Yaakov Hoffman, Jan Kizilhan, Amit Shrira, David Cheishvili, Moshe Syzf, Ari Zivotofsky

**Affiliations:** Bar-Ilan University, Ramat Gan, HaMerkaz, Israel; State University in Baden-Württemberg, Stuttgart, Baden-Wurttemberg, Germany; Bar-Ilan University, Ramat Gan, HaMerkaz, Israel; McGill University, Montreal, Quebec, Canada; EpiMedTech Global, Singapore, Singapore; Bar-Ilan University, Ramat Gan, HaMerkaz, Israel

## Abstract

Previous theories have linked exposure to trauma, and particularly trauma symptoms, to greater physical “wear and tear” that manifests as poorer health and metabolic syndrome. Very recent studies have shown that post-traumatic stress disorder (PTSD, which comprises, according to the ICD-11, three symptom clusters; intrusions, avoidance and arousal) longitudinally leads to more accelerated epigenetic aging. This study examines for the first time how complex-PTSD (CPTSD; which includes PTSD in addition to another three symptom clusters related to disturbances in self-organization; affect dysregulation, negative self-concept, and difficulties in forming and maintaining relationships) is linked with epigenetic accelerated aging. The sample comprised 94 female Yazidi women who had been held as sex slaves by ISIS and emigrated to Europe after their release ad who were resettled in Europe after their release from captivity were sampled (Mage 38.41±11.93, range 18-72.81; 63.2% female). Measures included demographic variables, PTSD and CPTD questionnaires along with salvia from which epigenetic aging was derived. Results show older epigenetic aging in those with CPTSD. Their epigenetic age was 4 years greater than ex-captives who did not display trauma symptoms. The data also revealed shorter telomere length for those with CPTSD. Although preliminary, these results demonstrate how complex trauma is associated with accelerated epigenetic mechanisms and address factors that speed up biological aging. The results have implications for normal aging, especially when one ages in the shadow of stress and trauma.

